# Integration of the metabolome and transcriptome reveals the mechanism of resistance to low nitrogen supply in wild bermudagrass (*Cynodon dactylon* (L.) Pers.) roots

**DOI:** 10.1186/s12870-021-03259-0

**Published:** 2021-10-21

**Authors:** Dandan Li, Jianxiu Liu, Junqin Zong, Hailin Guo, Jianjian Li, Jingjing Wang, Haoran Wang, Ling Li, Jingbo Chen

**Affiliations:** The National Forestry and Grassland Administration Engineering Research Center for Germplasm Innovation and Utilization of Warm-season Turfgrasses, Institute of Botany, Jiangsu Province and Chinese Academy of Sciences, Nanjing, 210014 Jiangsu Province China

**Keywords:** Bermudagrass, Low nitrogen nutrition, Metabolome, Transcriptome, Translation

## Abstract

**Background:**

Nitrogen (N) is an essential macronutrient that significantly affects turf quality. Commercial cultivars of bermudagrass (*Cynodon dactylon* (L.) Pers.) require large amounts of nitrogenous fertilizer. Wild bermudagrass germplasm from natural habitats with poor nutrition and diverse N distributions is an important source for low-N-tolerant cultivated bermudagrass breeding. However, the mechanisms underlying the differences in N utilization among wild germplasm resources of bermudagrass are not clear.

**Results:**

To clarify the low N tolerance mechanism in wild bermudagrass germplasm, the growth, physiology, metabolome and transcriptome of two wild accessions, C291 (low-N-tolerant) and C716 (low-N-sensitive), were investigated. The results showed that root growth was less inhibited in low-N-tolerant C291 than in low-N-sensitive C716 under low N conditions; the root dry weight, soluble protein content and free amino acid content of C291 did not differ from those of the control, while those of C716 were significantly decreased. Down-regulation of N acquisition, primary N assimilation and amino acid biosynthesis was less pronounced in C291 than in C716 under low N conditions; glycolysis and the tricarboxylic acid (TCA) cycle pathway were also down-regulated, accompanied by a decrease in the biosynthesis of amino acids; strikingly, processes such as translation, biosynthesis of the structural constituent of ribosome, and the expression of individual aminoacyl-tRNA synthetase genes, most of genes associated with ribosomes related to protein synthesis were all up-regulated in C291, but down-regulated in C716.

**Conclusions:**

Overall, low-N-tolerant wild bermudagrass tolerated low N nutrition by reducing N primary assimilation and amino acid biosynthesis, while promoting the root protein synthesis process and thereby maintaining root N status and normal growth.

**Supplementary Information:**

The online version contains supplementary material available at 10.1186/s12870-021-03259-0.

## Background

Nitrogen (N) is an essential macronutrient that is required for plant growth and development, and it strongly influences physiological parameters and performance. Plants often grow in N- limiting soil and need to apply a large amount of nitrogenous fertilizer. The N use efficiency of most plant species ranges from 30 to 50 % [1], and excessive N fertilizer leaks into the environment, causing global warming, soil acidification, groundwater pollution and other adverse consequences [2, 3]. Thus, it is very important to enhance plant N use efficiency under conditions of low N input. There are significant differences in N uptake and utilization among plant species. For example, modern wheat (*Triticum aestivum*) cultivars and maize (*Zea mays*) hybrids exhibit better growth than older accessions under N-limiting conditions [4]; modern rice (*Oryza sativa* L.) has lost the transcription factor *OsTCP19*, which is related to a high tillering response to N, but this transcription factor is prevalent in wild rice lines collected in natural habitats, most of which usually have low N content [5]. Significant differences in N uptake rate at low N concentrations were found among Kentucky bluegrass (*Poa pratensis* L.) genotypes [6]. As the most commonly used fertilizer for turfgrass, N significantly affects turf quality, including colour, density, recovery potential and stress resistance [7]. Utilizing high-quality wild germplasm of turfgrass collected from different natural habitats with diverse N content to improve the N use efficiency of turfgrass and reduce the requirement for N fertilizer input to turf have become a major goal of turfgrass researchers [8].

Bermudagrass (*Cynodon dactylon* (L.) Pers.) is widely used in lawns, sports turf, parks, golf courses, and tropical coral islands in tropical and subtropical regions [9, 10]. A great quantity of nitrogenous fertilizer is needed to establish and manage commercial cultivars of bermudagrass turf, which requires an N application of 3.6–9.8 g N m^2^wk^− 1^ [11]. Wild bermudagrass germplasm resources are very abundant and are widely distributed latitude of 19°02′-43°06′ N, 75°05′-122°02′ E, which are mainly distributed in the Yellow River basin and the southern area of China [12]. We collected a large number of wild germplasm resources of bermudagrass from regions in the above areas that have poor soil nutrition and diverse N distributions. We evaluated the collected wild germplasm resources and found that they showed significant differences in N accumulation and utilization [13]. However, the mechanisms underlying differences in N utilization by wild germplasm resources of bermudagrass remain unclear.

The physiological, cellular and molecular aspects of plants’ responses to low N nutrition have been studied in depth [14]. Roots are responsible for the active uptake minerals by plants [15]. Root growth and distribution grass-specific affects N uptake by perennial, warm-season grasses [16, 17]. Under N-limiting conditions, most plants increase their ability to acquire N by promoting root growth, and inducing the expression of high-affinity ammonium and nitrate transport systems [15, 18]. N starvation selectively down-regulates nitrate assimilation and amino acid metabolism in maize but has no influence on the expression of transcripts related to ammonium assimilation [19]. Ammonium is produced in plant cells not only as a result of primary nitrate assimilation but also by photorespiration and through the turnover of amino acids and proteins. Volatilization of ammonium to the environment can effectively be avoided into the environment by N remobilization under conditions of N limitation [20]. N remobilization increases in *Arabidopsis thaliana* plants that have adapted to long-term N deficiency [14]; however, total amino acid and protein contents are significantly reduced under N-limiting conditions [21]. In grass, the relative importance of mobilized N in growth was not significantly affected by low N nutrition [22]. In the initial step of protein synthesis, amino acids bind to tRNA through ester bonds. Protein synthesis is the most complex metabolic function in plants, and it mainly involves translation, a process that consumes approximately half of the energy that is required during rapid plant growth [23, 24]. In Arabidopsis, N-deprivation induces expression of most of the genes involved in amino acid biosynthetic pathways, amino acid tRNA synthetase, and protein synthesis [25].

N metabolism is closely linked to carbon (C) metabolism. N assimilation, in which inorganic N is incorporated into organic compounds, requires C skeletons, reducing power, and energy from C metabolism [19]. Analysis of differentially expressed genes and metabolites in rice using the Kyoto Encyclopedia of Genes and Genomes (KEGG) showed that N obviously affected C and N metabolism and amino acid metabolism [26]. Low-N-tolerant wild soybean (*Glycine max* (Linn.) Merr.) has a higher C/N ratio than common wild soybean at low N conditions [7]. Schlüter et al. [27] demonstrated that starch accumulates in maize under low N conditions; however, neither starch nor sucrose was found to accumulate in bermudagrass under low N conditions [28]. Xin et al. [29] performed an integrated analysis of the transcriptome and metabolome in rice and found that the tricarboxylic acid (TCA) cycle is enhanced to produce more energy and 2-ketoglutarate (2-OG) for N transport and assimilation under low N conditions. Current studies of plant adaptation to low N stress are mainly limited to investigating plant growth, biomass partitioning, N uptake, assimilation and remobilization and C metabolism [14, 21]. These studies have identified many N-responsive transcripts that are associated with the above processes, but many genes with unknown effects are also involved in aspects of plant metabolism under conditions of low N stress.

Wild germplasm resources in plants are important in the field of botany. N availability in Arabidopsis [14], rice [26, 29], maize [18, 27], barley (*Hordeum vulgare*, [30]), soybean [7] and sorghum (*Sorghum bicolor*, 15) has been investigated through integrated transcript and metabolite analysis. Cui et al. [31] reported the genome of a diploid *Cynodon* species, providing a preliminary genomic basis for understanding its adaptation to tropical and/or subtropical climates. However, there are almost no studies of the response of bermudagrass to low N nutrition that integrate transcriptomics, metabolomics and physiology. The purpose of this study is to reveal the mechanisms of adaptation of bermudagrass wild germplasm to low N nutrition. We used two bermudagrass wild accessions (one low-N-sensitive and one low-N-tolerant) to investigate plant growth, C and N accumulation and obtained metabolome and transcriptome profiles roots of the two wild bermudagrass accessions. This study provides a basis for the further use of high-quality wild bermudagrass germplasm resources and the breeding of low-N-tolerant bermudagrass cultivars.

## Methods

### Plant materials

Low-N-tolerant (C291) and low-N-sensitive (C716) accessions of wild bermudagrass that showed significant differences in N utilization under low N conditions in a previous study [13], were used in the experiments. The selected bermudagrass accessions were harvested from an experimental field (118°20’ E, 32°00’ N, mean annual precipitation: 1,106 mm, mean annual temperature: 15 °C) of National Main Warm Season Turfgrass Gene Bank at the Institute of Botany, Jiangsu Province and Chinese Academy of Sciences in China. As *Cynodon dactylon* is not endangered, collection of samples for scientific purposes was permitted by local legislation. Professor Shouliang Chen, taxonomy major, and Jianxiu Liu, turfgrass major of Institute of Botany, Jiangsu Province and Chinese Academy of Sciences, undertook the formal identification of the samples according to *flora of Reipublicae Popularis Sinicae* (Vol.10, No.1, 1990). Previous morphological and DNA analyses also confirmed the correct identification of the two accessions [32, 33].

### Growth conditions

The hydroponic experiment was performed in a greenhouse under a temperature regime of 30 °C/23 °C (day/night). Uniform sprigs with a stem node, each containing a lateral branch with three young expanded leaves, were harvested from our experimental field. The sprigs were planted on polyvinyl chloride (PVC) plates with 2 cm diameter holes, secured by a sponge, and kept in a greenhouse. Each PVC plate was placed in a plastic box (17 cm in diameter and 20 cm in height) with 2.5 L of modified Hoagland solution (1.2 mM Ca(NO_3_)_2_.4H_2_O, 1.6 mM KNO_3_, 0.5 mM (NH_4_)_2_SO_4_, 1 mM MgSO_4_.7H_2_O, 1 mM KH_2_PO_4_, 5 µM Fe-EDTA, 0.5 mM NaCl, 0.55 µM MnSO_4_.H_2_O, 0.0385 µM ZnSO_4_.7H_2_O, 2.35 µM H_3_BO_3_, 0.0065 mM H_2_MoO_4_, 0.0165 µM CuSO_4_.5H_2_O). An electric air pump was used to provide continuous ventilation for 10 h each day. The pH was maintained at 6.0 using 1 M HCl. The nutrient solution was changed every 3 days. After 20 days, sprigs with uniform growth were selected and clipped to a uniform length of 5 cm. We conducted preliminary experiments to determine the optimum and limiting N conditions for bermudagrass. Plants were grown at N concentrations ranging from 0 to 40 mM. At 5 mM and above, growth was similar for both accessions, while at 0.5 mM the difference in growth of the two accessions was most significant. Therefore, we used a nutrient solution containing 0.5 mM N (0.12 mM Ca(NO_3_)_2_.4H_2_O, 0.16 mM KNO_3_, and 0.05 mM (NH_4_)_2_SO_4_) for the low N treatment and a solution containing 5 mM N (1.2 mM Ca(NO_3_)_2_.4H_2_O, 1.6 mM KNO_3_, and 0.5 mM (NH_4_)_2_SO_4_) for the control. The differences in the potassium and calcium levels in low N nutrient solution were supplemented with K_2_SO_4_ and CaCl_2_, respectively. The plants were harvested after N treatment for 14 days and divided into shoots and roots.

To verify the results obtained in the hydroponic tests, a sand culture experiment was conducted using PVC tubes (20 cm in diameter and 40 cm in height) with trays at the bottom. These plants were maintained in the same greenhouse as were those used in the hydroponic experiment. Sprigs of the two accessions (C291 and C716) were planted in PVC tubes filled with clean sand and nutrient solutions identical to those used in the hydroponic experiment were added to the sprigs every three days. The plants were grown in the PVC tubes for approximately two months. The turfgrass was clipped weekly to a height of 3 cm in this experiment. We selected the same two N levels (0.5 mM and 5 mM) for use in the hydroponic experiment for the low N treatment. During this period, the shoots were clipped weekly to 3 cm height, and the clippings were collected and oven dried at 80 °C for 72 h. The plants were harvested after N treatment for two months and divided into clippings, verdure, rhizome and roots.

### Determination of soluble protein, soluble sugar, free amino acid, and starch content

The soluble sugar content and the free amino acids content were analysed by the Fales [34] and Shukla et al. [35] methods, respectively. The dried sample (0.1 g) was extracted in ethanol (80 %) for 30 min at 80 °C, and centrifuged at 3000 g, and the supernatant was collected. The extraction process was repeated three times. The volume of the collected supernatant was adjusted to 25 ml with ethanol (80 %). The supernatant (0.5 ml) was transferred to tubes, and 5 ml H_2_SO_4_-anthrone reagent was added. The mixture was immersed in a water bath at 90 °C for 15 min. The soluble sugar content was measured at 620 nm. Then, 1 ml chromogenic agent and 1 ml acetate buffer (pH = 5.4) were added to the extract, and the mixture was heated in a boiling water bath for 15 min. After cooling, 3 ml ethanol (60 %, v/v) was added. The free amino acid content was determined by measurement of the absorbance of the sample at 570 nm.

The soluble protein content was determined according to the Bradford [36] method. The root samples (0.5 g) were weighed and homogenized in sodium phosphate buffer (50 mM, pH 7.0). The homogenates were centrifuged at 4 000 × g (4 °C) for 10 min. The soluble protein content was measured by spectrophotometry at 595 nm. The starch content of the roots was determined by the methods described by Xie et al. [37].

### Metabolome analyses

The samples (60 mg) were ground, 400 µl of water and 200 µl of chloroform were added to each sample, and the mixture was centrifuged at 12,000 g at 4 °C for 10 min. Then, 80 µl of 15 mg/ml methoxylamine hydrochloride in pyridine was added to 300 µl of each supernatant. The mixture was incubated for 90 min at 37 °C. Then, 20 µl of n-hexane and 80 µl of BSTFA (with 1 % TMCS) were added, and the mixture was derivatized for 60 min at 70 °C. The samples were statically incubated at room temperature for 30 min before GC-MS analysis.

The derivatized sample was analysed using a gas chromatography system (Agilent 7890B, Agilent 5977 A MSD system, Agilent Technologies Inc., CA, USA). A DB-5MS fused-silica capillary column (30 m × 0.25 mm × 0.25 μm, Agilent J & W Scientific, Folsom, CA, USA) was used to separate the derivatives. Helium (> 99.999 %) at a constant flow rate of 1 ml/min was used as the carrier gas. The injection volume was 1 µl in splitless mode, and the temperature was maintained at 260 °C. The initial temperature of the oven was 60 °C; the temperature was then increased to 125 °C at a rate of 8 °C/min, to 210 °C at a rate of 4 °C/min, to 270 °C at a rate of 5 °C/min rate, and to 305 °C at a rate of 10 °C/min, and finally maintained at 305 °C for 3 min. The temperature of the MS quadrupole was set at 150 °C, and the ion source (electron impact) was set at 230 °C. The collision energy was 70 eV. The scan range was from 50 to 500 m/z. ChemStation software (version E.02.02.1431, Agilent, USA) to acquire and preprocess the data. Metabolites were annotated based on the Fiehn or NIST databases. Data transformation was conducted using log2 in Excel 2010, and the generated data matrix was imported into the SIMCA 14.0 software package (Umetrics, Umeå, Sweden). The different metabolites were selected by combing the p value obtained through a two tailed Student’s t-test of normalized peak area in different groups and the variable influence on projection (VIP) value obtained using the OPLS-DA model. Metabolites with P ≤ 0.05 and VIP ≥ 1.0 were considered significant differential metabolites. KEGG pathway in metabolites was performed by OE Biotech. Co., Ltd (Shanghai, China) (https://www.oebiotech.com/).

### Transcriptomic analysis

Total RNA was extracted using an RNAprep Pure Plant Kit (Tiangen, China). The integrity of the extracted RNA was verified on an Agilent 2100 Bioanalyser (Agilent Technologies, Palo Alto, CA, USA).The sample was analysed with RNA integrity number ≥ 7. The cDNA library construction and transcriptome sequencing were performed by OE Biotech. Co., Ltd (Shanghai, China) (https://www.oebiotech.com/) on an Illumina HiSeq X Ten platform, and paired-end reads were generated. The functions of unigenes were annotated based on public databases such as NCBI nonredundant (NR), SwissProt, KEGG pathway, Clusters of Orthologous Groups for eukaryotic complete genomes (KOG), and the GO database [38] using an E- value of 10^− 5^. The relationship between the SwissProt and GO terms was mapped for GO classification, based on the SwissProt annotation. The KEGG (http://www.kegg.jp/) [39] database was used to annotate potential unigene metabolic pathways.

### qRT-PCR validation

We randomly selected 10 genes for analysis by qRT-PCR. A qTOWER 2.2 Real-Time PCR system (Analytik Jena, Jena, Germany) was used to perform qRT-PCR assays. The primers used in qRT-PCR are listed in Table S1. The expression levels of the mRNAs were normalized to *EF-1α* (Cynodon) and were calculated by the 2^−ΔΔCt^ method [40].

### Accession numbers

The RNA-seq data generated in this research have been uploaded to the SRA database in NCBI under the accession number PRJNA693979 (SAMN17490887, SAMN17490888, SAMN17490889, SAMN17490890, SAMN17490891, SAMN17490892, SAMN17490893, SAMN17490894, SAMN17490895, SAMN17490896, SAMN17490897, SAMN17490898, SAMN17490899, SAMN17490900, SAMN17490901, SAMN17490902, SAMN17490903, SAMN17490904, SAMN17490905, SAMN17490906, SAMN17490907, SAMN17490908, SAMN17490909, and SAMN17490910).

### Statistical analysis

Each of the transcriptomic and metabolomic date points represents three biological replicates. Two-way analysis of variance was performed to compare dry weight, starch content and soluble sugar content, soluble protein content and free amino acid content between the accessions and between plants that received the N and control treatments using by the least significant difference (LSD) test at P < 0.05 in Statistical Product and Service Solutions software (SPSS Inc., Chicago, IL, USA).

## Results

### Effects of low N supply on bermudagrass growth

In the hydroponic experiment, the low N supply condition significantly affected plant growth. The shoot dry weight and plant dry weight decreased under the low N condition (Table 1); this decrease was less evident in accession C291 than in accession C716. Low N had no significant effect on the root dry weight of C291, but root dry weight was obviously reduced in C716 compared with the control.

Although some differences between the two accessions were observed in the sand culture experiment, the difference in their growth in response to low N nutrition was similar to that observed in the hydroponic experiment. Under low N conditions, the clipping dry weight, verdure dry weight and plant dry weight of C291 did not differ significantly from the control, but these parameters were obviously decreased in C716 compared with the control (Table 2). Low N had no significant effect on the root dry weight of C291, but root dry weight was decreased in C716 compared with that of the control.

**Table 1:** Influence of low N nutrition on biomass accumulation in the hydroponic experiment

Cultivars	Treatment	Shoot dry weight	Root dry weight	Plant dry weight
		(g. plant^− 1^)	(g. plant^− 1^)	(g. plant^− 1^)
C291	5 mM	25.18 ± 1.00 b	1.37 ± 0.09 ab	26.55 ± 1.09 b
	0.5 mM	20.29 ± 1.05 c	1.17 ± 0.21 ab	21.46 ± 1.13 c
C716	5 mM	28.76 ± 1.44 a	1.45 ± 0.06 a	30.21 ± 1.50 a
	0.5 mM	17.27 ± 1.62 d	1.12 ± 0.10 b	18.39 ± 1.61 d
	F_A_	0.14	0.04	0.14
	F_N_	114.39**	10.60*	107.87**
	F_A*N_	18.60**	0.64	17.12**

F_A_, F_N_, and F_A*N _refer to the F values of the accession, N treatment, and the interaction between the accession and N treatment, respectively. ** indicates significance at the level of P < 0.01; the letters after the number represent significant differences (*P* < 0.05).

**Table 2:** Influence of low N nutrition on biomass in the sand culture experiment

Cultivars	Treatment	Total clipping dry weight (mg.cm^− 2^)	Verdure dry weight (mg.cm^− 2^)	Rhizome dry weight (mg.cm^− 2^)	Root dry weight (mg.cm^− 2^)	Plant dry weight (mg.cm^− 2^)
C291	5 mM	32.06 ± 0.31 ab	29.08 ± 1.11 a	26.20 ± 1.42 a	6.81 ± 0.34 a	94.15 ± 1.97 a
	0.5 mM	28.72 ± 1.45 bc	27.29 ± 1.09 a	27.47 ± 1.44 a	7.14 ± 0.41 a	90.62 ± 2.32 a
C716	5 mM	32.70 ± 1.00 a	16.69 ± 1.37 b	20.16 ± 0.94 b	6.73 ± 0.24 ab	76.28 ± 2.42 b
	0.5 mM	25.36 ± 1.05 c	10.71 ± 1.12 c	22.09 ± 1.18 b	6.36 ± 0.57 b	64.52 ± 1.92 c
	F_A_	1.76	380.22**	32.92**	12.74*	276.26**
	F_N_	27.09**	27.31**	2.61	0.02	25.74**
	F_A*N_	3.8	7.97*	0.11	8.18*	9.00*

F_A_, F_N_, and F_A*N _refer to the F values of the accession, N treatment, and the interaction between the accession and N treatment, respectively. ** indicates significance at the level of P < 0.01; the letters after the numbers indicate significant differences (*P* < 0.05).

### Effects of low N supply on soluble protein content, free amino acid content, starch content and sugar content

**Fig. 1:**
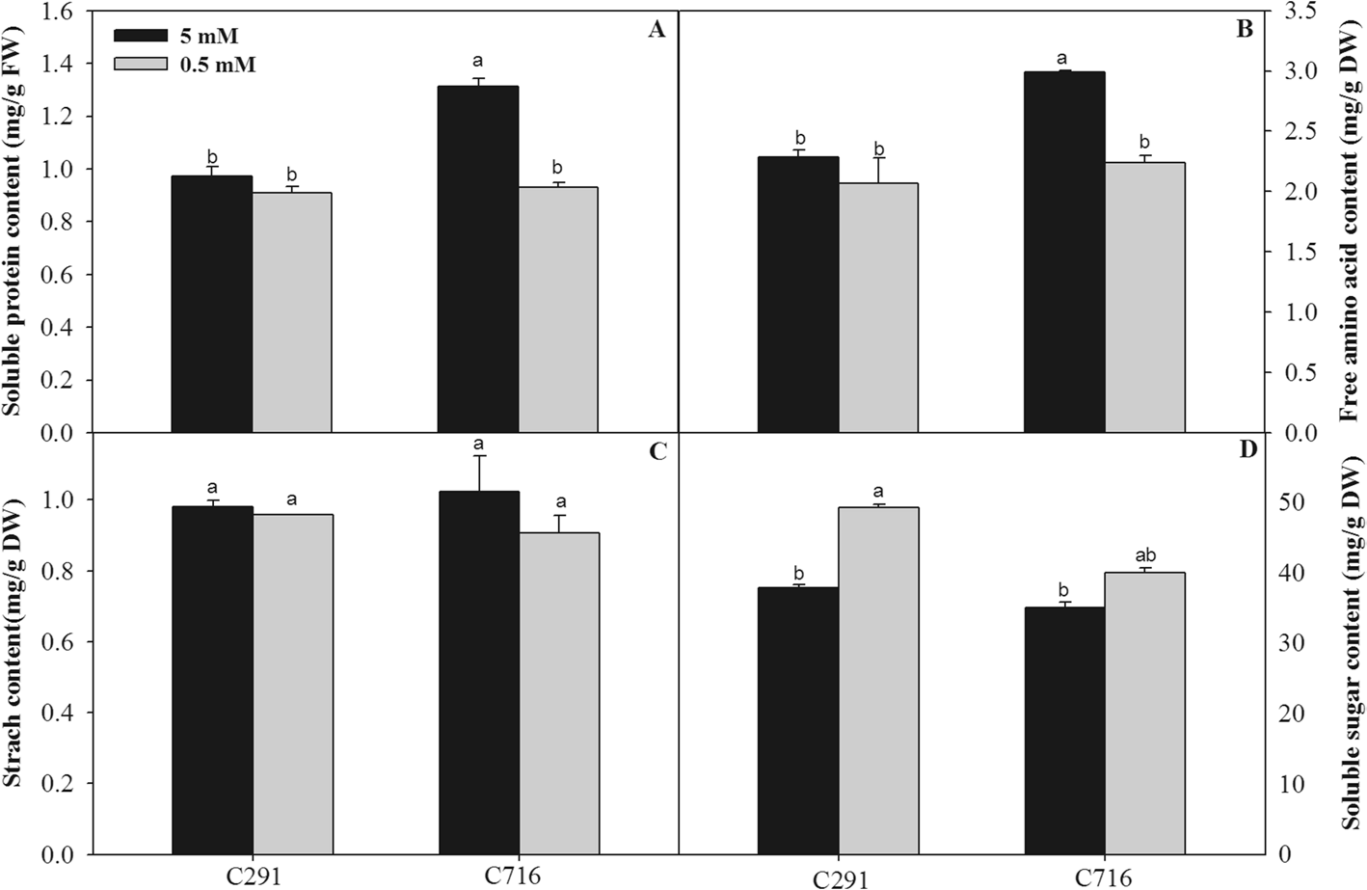
Physiological responses of the two accessions under low N conditions. The soluble protein content (**A**), the free amino acid content (**B**), the starch content (**C**) and the sugar content (**D**) of roots were measured. Lowercase letters represent significant differences between the accessions and between plants that received different N treatments (*P* < 0.05)

Low N availability had effects on the soluble protein content, free amino acid content, starch content and sugar content of the roots of the two accessions (Fig. 1). Under low N conditions, the root soluble protein content and the free amino acid content of C291 were not significant different from those in the control, but these parameters were significantly decreased in C716. Under low N conditions, the root starch content of the two accessions was unchanged, but the root soluble sugar content increased compared that of the control; this increase was less pronounced for C716 than for C291.

### Effects of low N supply on the metabolite profiles of bermudagrass roots

**Fig. 2:**
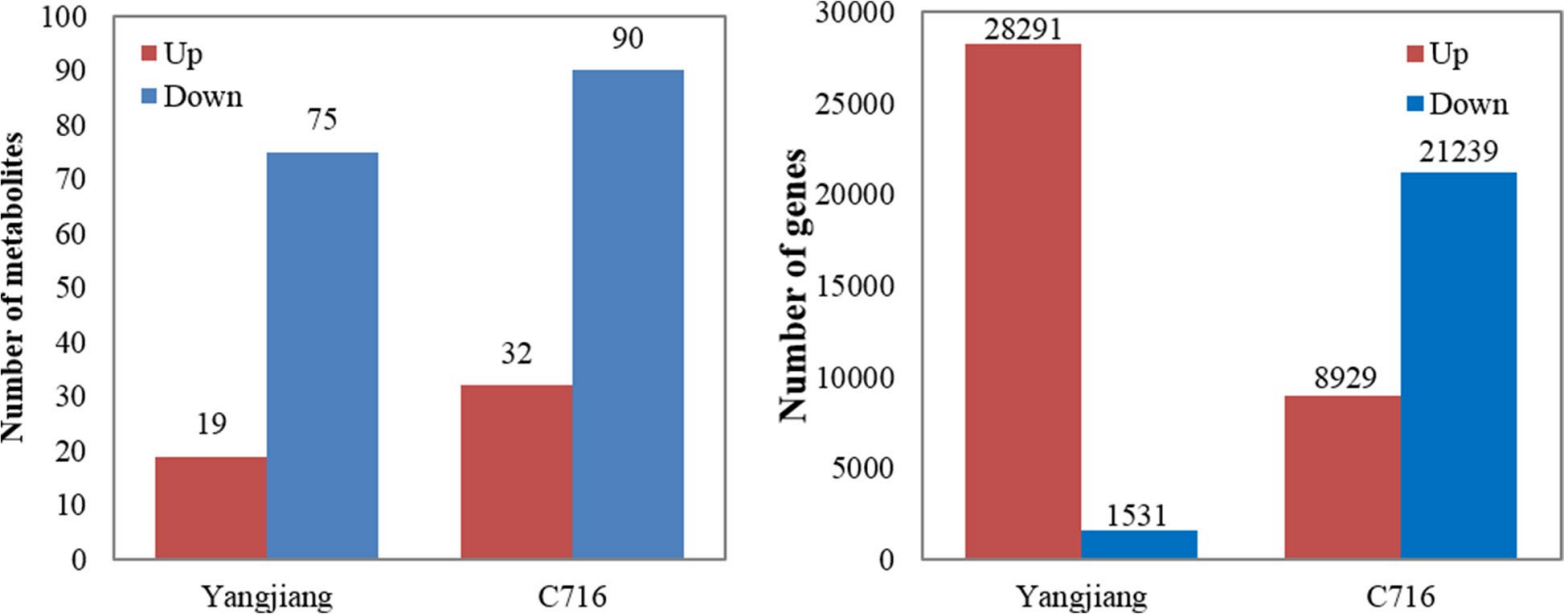
The numbers of different metabolites (**A**) and genes (**B**). The results included data up-regulated and down-regulated separately under low versus control N conditions. The bars show the means of the values obtained for three biological replicates

To comprehensively and systematically analyse the effect of low N nutrition on metabolites, we analysed the nontargeted metabolites by GC-MS. Under low N conditions, the number of metabolites whose concentrations increased was lower than the number of reduced metabolites whose concentrations decreased for both C291 and C716 separately compared with the control (Fig. 2 A). In C291, 94 metabolites were differentially accumulated compared with the control, and in C716 122 metabolites were differentially accumulated compared with the control at low N conditions. The numbers of up- and down-regulated metabolites compared were lower in C291 than in C716.

**Fig. 3:**
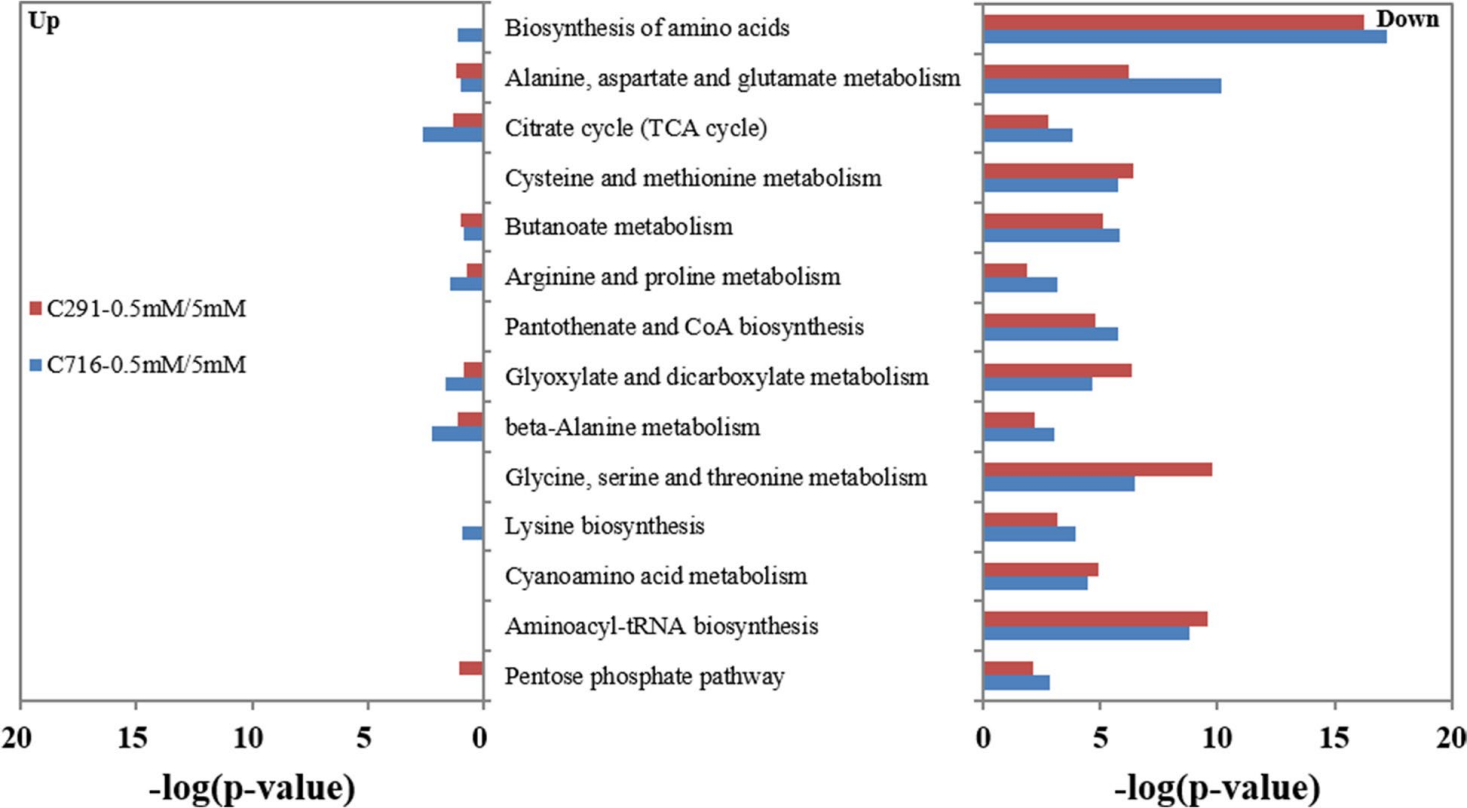
KEGG analysis of the differentially metabolites regulated by low versus control N conditions

The functions of different metabolites associated with different biological pathways were analysed using the KEGG (Fig. 3). In all pathways, the up-regulated metabolites were significantly less abundant than the down-regulated metabolites for both accessions under low versus control N conditions. The down-regulated metabolites were enriched in the ‘biosynthesis of amino acids’, ‘glycine, serine and threonine’, ‘alanine, aspartate and glutamate metabolism’ and ‘aminoacyl-tRNA biosynthesis’ pathways. In particular, ‘biosynthesis of amino acids’ process was down-regulated to a lesser extent in C291 than in C716, whereas ‘glycine, serine and threonine’ process was down-regulated more in C291 than in C716. Furthermore, the pathway ‘alanine, aspartate and glutamate metabolism’ was down-regulated less in C291 than in C716 compared with the control N level under low N levels.

### Effects of low N supply on the transcriptome profiles of bermudagrass roots

The expression profiles of bermudagrass were analysed by RNA-sEq. At low N levels, the number of differentially expressed genes compared with the control was lower in C291 than in C716 (Fig. 2B). In C291 under low N conditions, the number of up-regulated genes was significantly higher than the number of down-regulated genes, while the opposite was true in C716. In addition, we found that qRT-PCR and the transcriptome data were highly consistent at the gene expression levels (Fig. S1). The R-square value of the 10 validated genes was 0.94, indicating that the transcriptome data were reliable.

**Fig. 4:**
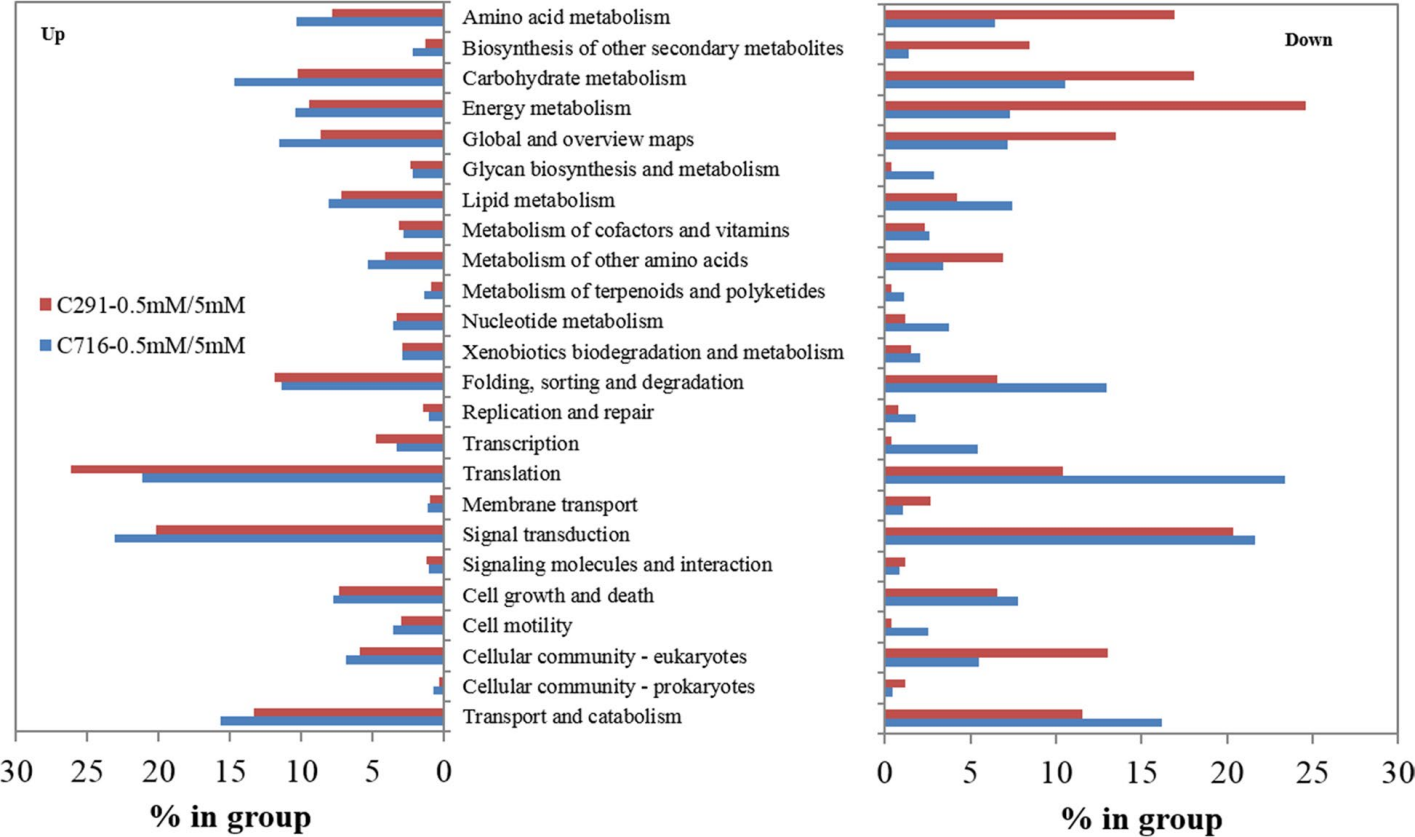
KEGG analysis of the differentially transcripts regulated at low versus control N conditions

KEGG enrichment analysis of the differentially up-regulated transcripts showed that they were significantly enriched in the ‘translation’, ‘signal transduction’ and ‘carbohydrate metabolism’ pathways; transcripts related to ‘amino acid metabolism’, ‘carbohydrate metabolism’, ‘energy metabolism’, ‘translation’, and ‘signal transduction’ tended to be down-regulated (Fig. 4). The down-regulated transcripts in C291 were enriched in the ‘amino acid metabolism’, ‘carbohydrate metabolism’, and ‘energy metabolism’ pathways were significant more than those in C716. Strikingly, under low versus control N levels, ‘translation’ was the most significantly up-regulated biochemical pathway in C291, while it was the most significantly down-regulated pathway in C716.

**Fig. 5:**
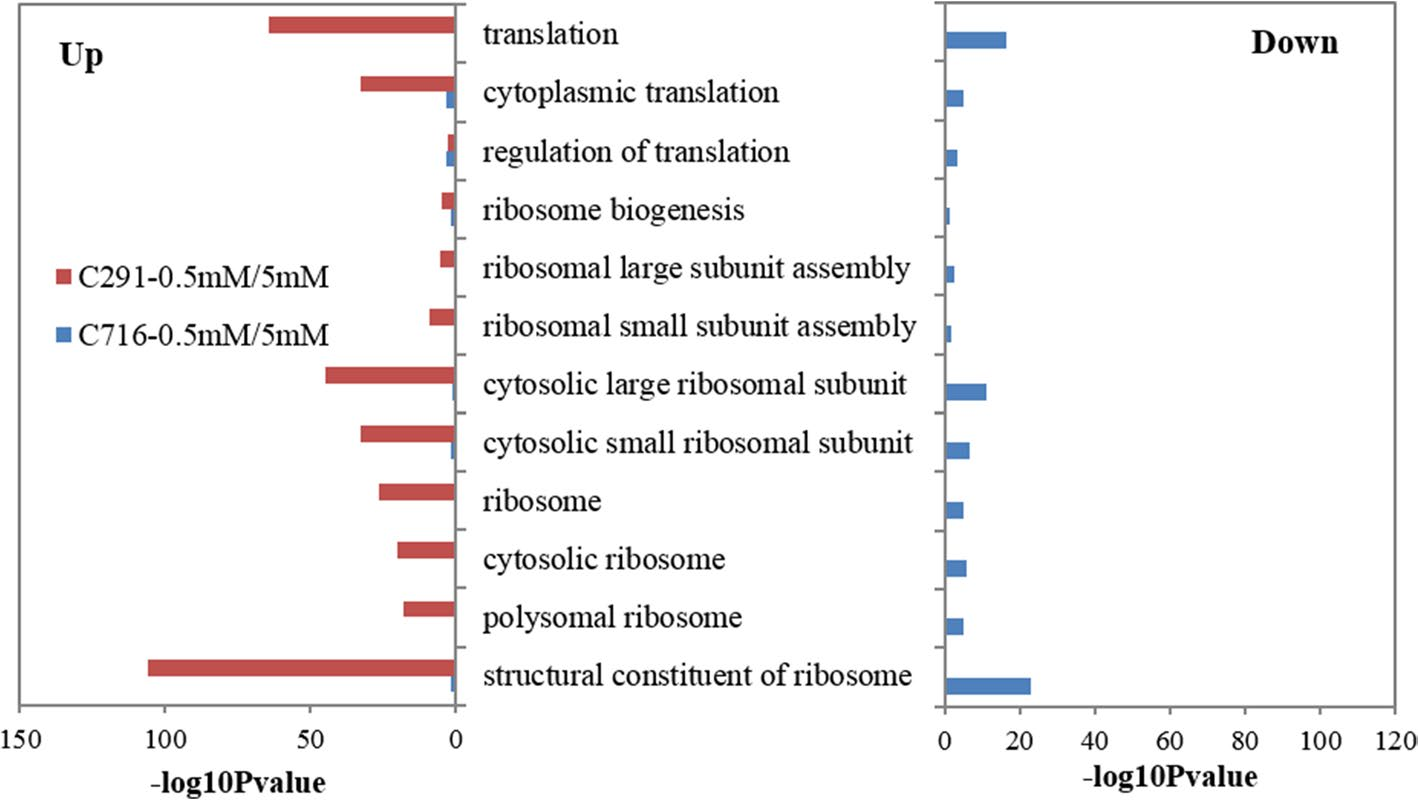
GO categorization analysis of the differentially transcripts related to translation under low versus control N conditions

To analyse the functions of the transcripts related to the ‘translation’ process that were differentially expressed in response to low N supply, GO enrichment analysis was conducted. A summary of the GO categories related to ‘translation’ is listed in Fig. 5. Under low versus control N conditions, transcripts related to protein synthesis with the exception of ‘regulation of translation’ were significantly up-regulated in C291, while they were down-regulated in C716 (Fig. 5). Furthermore, the transcripts that were up-regulated in C291 were most enriched in ‘structural constitute of ribosome’, ‘translation’, and ‘cytosolic large ribosomal subunit’, while in C716 down-regulated transcripts were more enriched in these GO terms.

### Effects of low N supply on N and C metabolism in bermudagrass roots

**Fig. 6:**
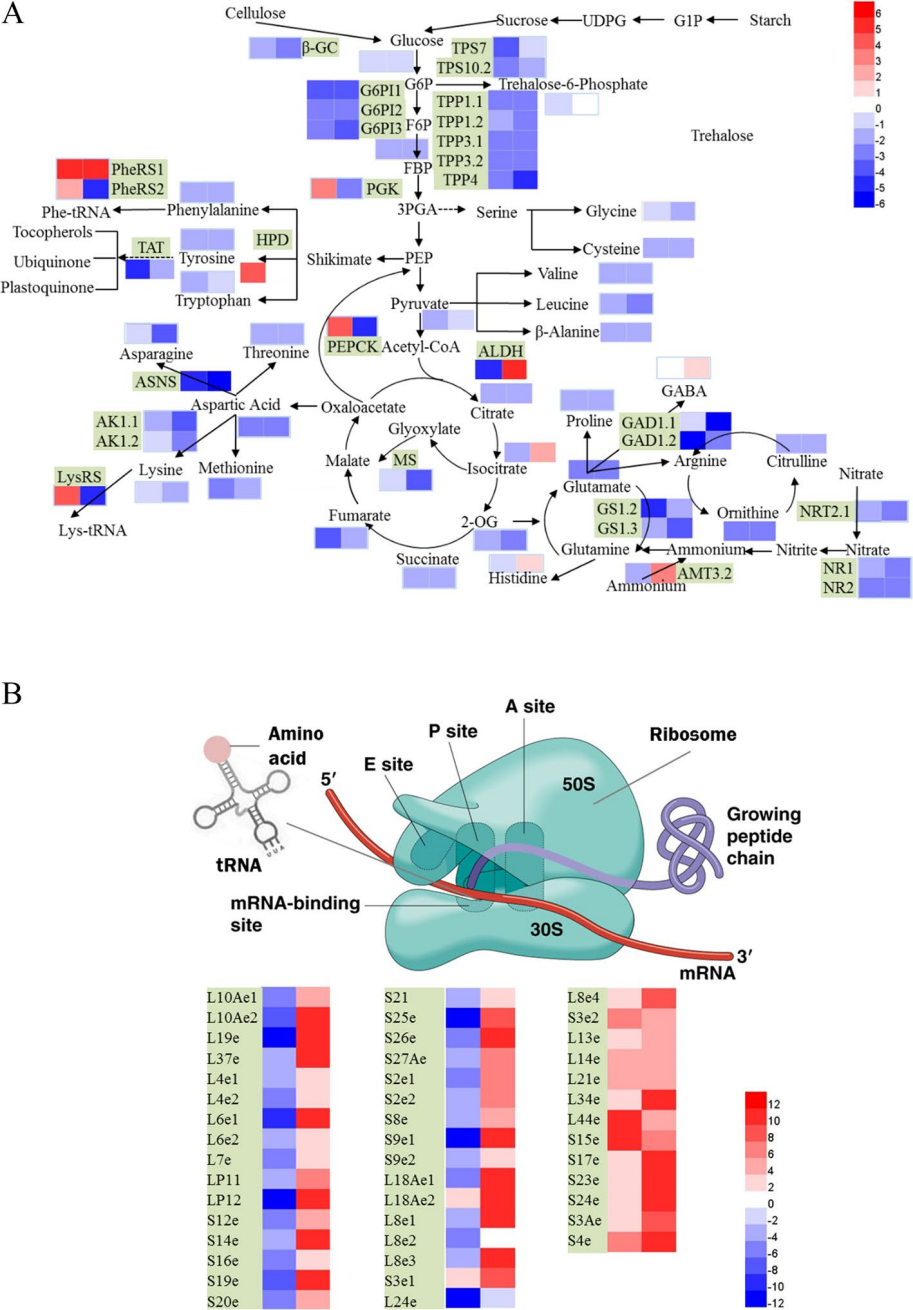
Changes in root metabolic processes (**A**) and protein synthesis (**B**) in the two accessions under low versus control N levels. The log2 ratios of the genes and metabolites are shown and the colour key is presented at the right of the figure. Black characters with a light green background represent genes, and black characters with no background represent metabolites. From left to right, the two squares illustrate changes in metabolites and genes in C291 and C716 under low versus control N conditions

N availability affected the C and N compounds present in bermudagrass roots, as shown by its effects on soluble sugar content and free amino acid content (Fig. 1). The results of transcriptome and metabolome analysis were consistent; they showed that N uptake, assimilation, amino acid metabolism, aminoacyl-tRNA biosynthesis, glycolysis, and the TCA cycle differed significantly under low versus control N conditions (Fig. 6 A).

The transcripts of genes associated with N uptake and assimilation in the roots of the two bermudagrass accessions differed under low N conditions (Fig. 6 A). The ammonium transporter gene *AMT3.2* was up-regulated in C716, but down-regulated in C291; however, the nitrate transporter gene *NRT2.1* was down-regulated more in C716 than in C291 at low versus control N conditions. The nitrate assimilation genes *NR1* and *NR2* and the ammonium assimilation genes *GS1.2* and *GS1.3* were down-regulated in both accessions; the metabolite glutamate was correspondingly down-regulated in both accessions compared with the control at low N levels. The *NR1* and *GS1.3* genes were down-regulated less in C291 than in C716 under low versus control N conditions.

Other than γ-aminobutyrate (GABA) and histidine, most metabolites related to amino acid metabolism were down-regulated in both accessions under low versus control N conditions (Fig. 6 A). GABA and histidine were up-regulated in C716, but did not differ significantly from the control in C291 under at low versus control N conditions. At low N levels, the metabolites asparagine, aspartate, glycine, leucine, ornithine, serine, and threonine were down-regulated less in C291 than in C716, while β-alanine, cysteine, methionine, phenylalanine, proline, tryptophan, and tyrosine were down-regulated to a greater extent in C291 than in C716. Consistent with these findings, the asparagine synthase *ASNS*, and the aspartate kinase *AK1.1* and *AK1.2* genes were down-regulated to a lesser extent in C291 than in C716, while the glutamate decarboxylase *GAD1.1* gene down-regulated to a greater extent in C291 than in C716. Furthermore, the aldehyde dehydrogenase (NAD^+^) *ALDH* gene was down-regulated in C291, while it was up-regulated in C716 at low versus control N conditions. In addition, the phenylalanyl-tRNA synthetase gene PheRS2 and the lysyl-tRNA synthetase gene LysRS were up-regulated in C291, but down-regulated in C716 under low versus control N conditions; in fact, most of the genes encoding ribosomal proteins required for protein synthesis were up-regulated in C291 but down-regulated in C716 (Fig. 6B).

Amino acid metabolism and primary N assimilation require energy and C skeletons provided by glycolysis and the TCA cycle (Fig. 6 A). Under low N conditions, 11 and 13 differentially abundant metabolites and genes respectively, were identified. The metabolites involved in glycolysis, including pyruvate, fructose-6-phosphate (F6P), and glucose-6-phosphate (G6P) were down-regulated more in C291 than in C716 at low versus control N levels; moreover, genes related to glycolysis, including the glucose-6-phosphate 1-epimerase (*G6PI1*) and phosphoglycerate kinase (*PGK*) genes were down-regulated more in C291 than in C716. The metabolites participating in the TCA cycle, including citrate, 2-OG, succinate and fumarate, were down-regulated in the two accessions under low- versus control-N conditions. In particular, metabolite isocitrate was down-regulated in C291 but up-regulated in C716. Isocitrate is converted to 2-OG by isocitrate dehydrogenase or decomposed into succinate and glyoxylate under the action of glyoxysomal isocitrate lyase; glyoxylate is further converted to malate by malate synthase in glyoxysome. 2-OG, as a C skeleton involved in NH_4_^+^ assimilation that serves as a precursor of glutamate, was down-regulated less in C291 than in C716; the malate synthase gene *MS* in glyoxysome was also down-regulated less in C291 than in C716, suggesting that the down-regulation of NH_4_^+^ assimilation and of the conversion of glyoxylate to malate in glyoxysome led to the accumulation of isocitrate in C716 under low N conditions. The above results indicated that glycolysis and TCA cycle processes were inhibited to produce more energy and C skeletons for primary N assimilation and amino acid biosynthesis under low N conditions.

Starch, cellulose, and some disaccharides are involved in glycolysis pathway and in the TCA cycle after conversion into glucose. Here, starch content showed no significant difference from the control for either of two accessions at low N conditions (Fig. 1); the β-glucosidase (E3.2.1.21) gene associated with cellulose metabolism was down-regulated more in C291 than in C716. Trehalose (a-D-glucopyranosyl a-D-glucopyranoside) is a disaccharide composed of two glucose molecules. In our study, trehalose-6-phosphate synthase (EC 2.4.1.15) genes *TPS7* and *TPS10.2* were down-regulated in the two accessions, and the *TPS7* and *TPS10.2* genes were down-regulated more in C291 than in C716. Correspondingly, the level of trehalose-6-phosphate (T6P) decreased more in C291 than in C716 at low versus control N levels (Fig. 6 A). The trehalose catabolism genes *TPP1.1*, *TPP1.2*, *TPP3.1*, *TPP3.2*, and *TPP4* were down-regulated at the transcript level; the transcript levels of the *TPP1.2* and *TPP4* were down-regulated more in C291 than in C716 under low versus control N levels.

## Discussion

N is an essential component of amino acids, amides, nucleotides, nucleic acids, pigments, and secondary metabolites [41]. Plants often encounter limiting levels of N in their natural habitats [5, 14]. In this study, we used two wild bermudagrass with different N tolerances collected from different habitats and found that they exhibited significant differences in their growth, physiology, metabolome and transcriptome aspects in responses to low N supply.

Under N limiting conditions, the ability of plants to obtain N is generally increased by promoting root growth relative to shoot growth [42] and by inducing the expression of high- affinity transporters of nitrate and ammonium [4]. In low-N-tolerant sorghum, high-affinity nitrate transporters are more abundant than they are in low-N-sensitive genotypes [15]. In our study, the shoot dry weights of the two accessions were indeed reduced compared with that of the control, while the root growth of the two accessions showed significant differences at low N levels; root dry weight reduced in the low-N-sensitive accession C716, while it showed no significant difference from the control in the low-N-tolerant accession C291 in either the hydroponic experiment (Table 1) or the sand culture experiment (Table 2). This is consistent with a report by Li et al. [13] that low-N-tolerant groups have higher relative root dry weight; in our study, nitrate transporter gene *NRT2.1* was down-regulated to a lesser extent in C291 than in C716 compared with the control under low N conditions (Fig. 6 A). After absorption by the roots, NO_3_^−^ is assimilated to NH_4_^+^ through the action of nitrate reductase (NR) and nitrite reductase (NIR) [43]; NH_4_^+^ can be converted into glutamate through the glutamine synthetase (GS) / glutamate synthase (GOGAT) cycle [44, 45], which also requires 2-OG as a C skeleton. In general, the expression and activity of enzymes that act in N assimilation are closely related to the N status of plant tissue and are reduced when N is limited [4, 14]. In maize, nitrate reduction is down-regulated while ammonium assimilation related transcripts are not affected by N starvation [19]. Here, the transcript levels of *NR1* and *GS1.3* were down-regulated more in C716 than in C291, and the 2-OG content of C716 correspondingly decreased more than that of C291 compared with that of the control at low N levels (Fig. 6). The above results suggest that primary N assimilation in roots was inhibited by low N, and that N assimilation decreased less in C291 than in C716.

In general, plants respond to N stress by reducing the synthesis of nitrogenous compounds, such as amino acids [4]. Here, KEGG analysis of differentially expressed transcripts and metabolites showed that they were significantly enriched in ‘amino acid metabolism’, which was down-regulated in both accessions (Figs. 3 and 4), as was found in many other studies conducted in rice [29] and maize [19]; however, the root free amino acid content of C716 decreased at low N conditions, while that of C291 showed no significant difference from the control. Furthermore, most amino acids were down-regulated, although individual amino acids such as GABA and histidine showed different responses to low N stress according to our metabolite analysis (Fig. 6 A). GABA accumulation has been found to be a common response to many environmental constraints, including salt [46, 47], drought [48], heat [49] and N stress [50]. GABA levels increase in plants concomitant with restrictions on glutamine synthesis and protein synthesis [51] and imbalances in C/N [50]. GABA levels were shown to be reduced in rice under low N conditions [26]. In the current study, GABA was significantly up-regulated in C716 under low N conditions, while in C291 it did not differ from the control (Fig. 6 A). Glutamate supplies amino group to many other amino acids. Here, glutamate, proline, and ornithine were all down-regulated compared with the control under low N conditions. Asparagine is generally used for N storage and is characterized by a high N:C ratio [19]. Here, in C291, asparagine, lysine and threonine, and transcripts of *ASNS*, *AK1.1* and *AK1.2* were less affected than those in C716 under low N conditions. These results indicate that amino acid metabolism in C291 was less affected by low N supply than that in C716.

Low N nutrition has far-reaching consequences on the expression of downstream genes related to N utilization, including the genes involved in protein synthesis. Proteins are the main drivers of cell function, and their synthesis is mainly accomplished by translation. N deprivation induces the expression of many genes related to protein synthesis independent of amino acid changes in Arabidopsis [25]. It is striking that the differentially expressed transcripts in our current study were significantly enriched in ‘translation’; transcripts related to this process were up-regulated to a significantly higher degree in C291 than in C716 and were down-regulated to obviously lower levels in C291 than in C716 (Fig. 4), indicating that the translation process was enhanced in low-N-tolerant accessions C291 under low N conditions. Translation is a basic biological process that transforms nucleotide sequences into amino acid sequences; it includes amino-tRNA synthesis, the assembly of polypeptides, and folding and processing of polypeptides [23]. Translation is performed by a ribonucleoprotein complex called the ribosome, which is consists of a large and a small subunit [24, 52]. Here, we specifically analysed GO terms related to ‘translation’ and found that transcripts related to ‘translation’ except those related to ‘regulation of translation’, were significantly up-regulated, while they were all obviously down-regulated in C716 under low versus control N conditions (Fig. 5). The initiation of protein biosynthesis requires the synthesis of aminoacyl-tRNA [53]. Here, the differentially abundant metabolites were also enriched in ‘aminoacyl-tRNA biosynthesis’ (Fig. 3); moreover, the phenylalanyl-tRNA synthetase gene *PheRS2* and the lysyl-tRNA synthetase gene *LysRS* were significantly up-regulated in C291, while they were obviously down-regulated in C716 under low versus control N conditions (Fig. 6A). Furthermore, most of the genes associated with ribosomes in protein synthesis were significantly up-regulated in C291, but obviously down-regulated in C716 (Fig. 6B). As a consequence, the protein content of C291 roots did not differ from that of the control, whereas the protein content of C716 roots significantly reduced, which suggesting that low-N-tolerant accessions in bermudagrass maintain root protein content through promoting the translation process as a means of adaptation to low N stress. Protein synthesis was stimulated in low-N-tolerant bermudagrass together with down-regulated amino acid biosynthesis; this may be related to improve N remobilization and increase the allocation of amino acids to roots under low N stress [20].

Glycolysis and the TCA cycle supply energy, reducing equivalents and C skeletons for N metabolism [14]. Here, KEGG analysis of differentially abundant metabolites compared with those of the control showed that the TCA cycle was down-regulated in bermudagrass roots at low N levels (Fig. 3); furthermore, KEGG terms were enriched in ‘carbohydrate metabolism’ and ‘energy metabolism’ for the two accessions, and these processes were down-regulated more in C291 than in C716 (Fig. 4). Moreover, the genes *G6PI1* and *PGK*, which are associated with the glycolysis pathway were down-regulated more in C291 than in C716, and the levels of the metabolite G6P, F6P, and pyruvate were lower in C291 than in C716, indicating that glycolysis was more affected in C291 than in C716 by a low N supply. The organic acid pool was generally reduced due to N limitations in bermudagrass roots; however, the response of the two accessions was different; in C291, the *MS* gene and the metabolites of citrate and 2-OG were down-regulated less than they were in C716 at low-versus control N levels (Fig. 6 A). This is similar to previous results in maize [19] and rice [54]. Under N limiting conditions, when the demand for organic acids for amino acid synthesis decreases, the transformation of photosynthetic products shifts to other final products; therefore, the biosynthesis of starch and sucrose usually increase [4, 55]. In our current study, soluble sugar content indeed increased in both accessions compared with those of the control at low N levels (Fig. 1), in agreement with many similar studies [14, 56]. In addition, the accumulation of trehalose and T6P and overexpression of *TPS* [57] in plants significantly retarded growth under various types of stress [58]. Here, the metabolite T6P and the *TPS7* and *TPS10.2* genes were down-regulated more in C291 than in C716 (Fig. 6 A), and the growth of C291 was correspondingly less affected by low N stress than C716 (Table 1).

## Conclusions

The growth, physiological, transcript and metabolite analyses reported here suggest that the C291 low-N-tolerant wild germplasm of bermudagrass is better adapted than the C716 variety to efficiently utilize N under N-limiting conditions. The main differences between the accessions (low-N-tolerant and low-N-sensitive) were in the magnitude of the response to low N supply. The growth of the low-N-tolerant accession C291 was less inhibited by low N supply than that of C716. When N was limited, C291 displayed fewer reductions in N acquisition, primary N assimilation and amino acid biosynthesis than did C716. Glycolysis and the TCA cycle were also down-regulated accompanied by a decrease in the biosynthesis of N-containing amino acid. Strikingly, most of the transcripts were enriched for the term ‘translation’, which relates to protein synthesis; furthermore, terms related to the translation machinery, such as ribosomes, cytosolic large ribosome subunits and structural constituents of ribosomes, were all up-regulated in C291 but down-regulated in C716; moreover, individual aminoacyl-tRNA synthetase genes and most of the genes involved ribosome for protein synthesis were up-regulated in C291, but down-regulated in C716; as a consequence, the protein content of C291 roots did not differ from that of the control, but it was significantly reduced in C716. This suggests that low-N-tolerant accessions of bermudagrass maintain root growth and their N status by promoting protein synthesis process in a way that allows them to adapt to low N stress. These results contribute to a deeper understanding of the physiological and molecular mechanisms involved in the adaptation of wild bermudagrass germplasm to low N environments, and provide a basis for the application of these mechanisms to low-N-tolerant bermudagrass breeding.

## Supplementary Information

**Additional file 1: **

## Data Availability

Raw Illumina sequence data were deposited in the National Center for Biotechnology Information (NCBI) and be accessed in the sequence read archive (SRA) database (https://www.ncbi.nlm.nih.gov/sra). The accession number is PRJNA693979 (https://www.ncbi.nlm.nih.gov/sra/PRJNA693979), which includes 24 accession items (SAMN17490887- SAMN17490910). All data generated or analysed during this study are included in this published article and supplementary information files.

## Abbreviations

N: Nitrogen
C: Carbon
KEGG: Kyoto encyclopedia of genes and genomes
GO: Gene ontology
DEGs: Differentially expressed unigenes
TCA: Tricarboxylic acid
PPP: Pentose phosphate pathway
PVC: Polyvinyl chloride
VIP: Variable influence on projection
GABA: γ-aminobutyrate
PGK: Phosphoglycerate kinase
TPS: Trehalose-6-phosphate synthase
TPP: Trehalose-6-phosphate phosphatase
T6P: Trehalose-6-phosphate
2-OG: 2-oxoglutarate
GS: Glutamine synthetase
GOGAT: Glutamate synthase
