# Supplementary material for: Integration of the metabolome and transcriptome reveals the mechanism of resistance to low nitrogen supply in wild bermudagrass (Cynodon dactylon (L.) Pers.) roots
Source: BMC Plant Biol. 2021 Oct 21;21:480. doi: 10.1186/s12870-021-03259-0 (PMC8532362; doi:10.1186/s12870-021-03259-0)
Supplement: Supplementary file 1 — Additional file 1: [file 12870_2021_3259_MOESM1_ESM.docx]

Fig. S1. Correlation analysis between transcriptome and qRT-PCR results.

Table S1 The sequences for primers used in qRT-PCR

| **Num** | **Gene Symbol** | **Forward primer(5->3)** | **Reverse primer(5->3)** |
| --- | --- | --- | --- |
| **1** | EF-1α bermudagrass | TGTCCCTGTCGGTCGTGTT | AGCCTCATGGTGCATCTCA |
| **2** | TRINITY_DN146875_c0_g1_i1_1 | AATCAGAGCAAGTACACCTATG | CTTCACCGGAGATGACCA |
| **3** | TRINITY_DN30480_c0_g1_i1_4 | TCTCTGTCATCAAGGAACTGC | CAACTGATCCAACAAACCTGAA |
| **4** | TRINITY_DN155149_c0_g1_i1_4 | AAGAAGTCGGAGCTGGAGA | ATTATTAGCAGACGCCGTTG |
| **5** | TRINITY_DN59781_c0_g1_i1_3 | AGGATGTTGTCAGTGATGTTC | TGTCTCAAATGTGGTCAACG |
| **6** | TRINITY_DN139224_c0_g2_i3_1 | TCTGAATGAGCCTGGGACA | GTAGCTCTAGCGAACATGC |
| **7** | TRINITY_DN134698_c0_g1_i1_4 | GACCTCTCCACGGGAAAC | GTGAAGAGGAAGGCGAGC |
| **8** | TRINITY_DN139237_c0_g1_i1_4 | TCTATTGTGCGTTGAGGGTC | ACCGTCGATGTGTTTGGTA |
| **9** | TRINITY_DN27246_c0_g1_i1_5 | TGGATTCTTCCCAGATCGTC | ACAGTCGGATGGAGAATACC |
| **10** | TRINITY_DN26871_c0_g1_i1_5 | AAACCAATCCCAATGCGA | CTCAAGACCTTCCCAGTAGTAT |
| **11** | TRINITY_DN60279_c0_g1_i4_3 | ACAGTGAAGTCATCCTTTGTC | CGTAGCAGTCGCACATAAC |
